# Species Composition, Ecological Preferences, and Chromosomal Polymorphism of Malaria Mosquitoes of the Crimean Peninsula and the Black Sea Coast of the Caucasus

**DOI:** 10.3390/insects16040367

**Published:** 2025-04-01

**Authors:** Anton V. Moskaev, Anna G. Bega, Ilya I. Brusentsov, Anastasia N. Naumenko, Dmitriy A. Karagodin, Vladimir N. Razumeiko, Boris V. Andrianov, Irina I. Goryacheva, Elizaveta Y. Lee, Vladimir I. Panov, Igor V. Sharakhov, Maria V. Sharakhova, Mikhail I. Gordeev

**Affiliations:** 1 Laboratory of Experimental Biology and Biotechnology, Scientific and Educational Center in Chernogolovka, Federal State University of Education, Moscow 105005, Russia; anton-moskaev@yandex.ru (A.V.M.); anni.miya@gmail.com (A.G.B.); iigoryacheva@mail.ru (I.I.G.); lilizavetau@mail.ru (E.Y.L.); sobol4ek95@yandex.ru (V.I.P.); 2 Analytical Laboratory for Environmental Monitoring, Vernadsky Russian State University of National Economy, Balashikha 143907, Moscow Region, Russia; 3 Laboratory of Insect Genetics, Vavilov Institute of General Genetics, Russian Academy of Sciences, Moscow 119991, Russia; andrianovb@mail.ru; 4 Laboratory of Cell Differentiation Mechanisms, The Federal Research Center, Institute of Cytology and Genetics, Novosibirsk 630090, Russia; brusentsovi@gmail.com (I.I.B.); karagodin@bionet.nsc.ru (D.A.K.); msharakh@vt.edu (M.V.S.); 5 Department of Entomology, The Fralin Life Sciences Institute, Virginia Polytechnic Institute and State University, Blacksburg, VA 24061, USAigor@vt.edu (I.V.S.); 6 Department of Ecology and Zoology, Vernadsky Crimean Federal University, Simferopol 295007, Russia; razumeiko@gmail.com; 7 Department of Genetics and Cell Biology, Tomsk State University, Tomsk 634050, Russia; 8 Department of General Biology and Ecology, Federal State University of Education, Moscow 105005, Russia

**Keywords:** *Anopheles*, the Crimean Peninsula, Black Sea coast of Caucasus, distribution, breeding sites, chromosomal polymorphism

## Abstract

In this study, we sampled malaria mosquito larvae in natural and artificial breeding places to study the geographical distribution, ecological preferences, and chromosomal variability of different species of the genus *Anopheles* in the territory of the Crimean Peninsula and the Black Sea coast of the Caucasus. Species were diagnosed using a combination of morphological, cytogenetic, and molecular markers. The ecological conditions of the larval habitats, such as dissolved oxygen content in the water, acidity, salinity, and temperature, were measured. Seven species of malaria mosquitoes were identified in the pool of 2229 individual mosquitoes collected at 56 breeding sites, including *An. atroparvus*, *An. claviger*, *An. daciae* (formerly identified as *An. messeae* s. l.), *An. hyrcanus*, *An. maculipennis* s. s., *An. plumbeus*, and *An. melanoon*. The previously recorded species of *An. algeriensis*, *An. messeae* s. s., *An. sacharovi*, and *An. superpictus* were not found in this study. *Anopheles maculipennis* was dominant in typical anophylogenic water bodies. *Anopheles plumbeus*, which used to breed mainly in tree holes in coastal forests, has spread to urban settlements along the Black Sea coast and breeds in artificial containers. Chromosomal polymorphism was studied and found in *An. atroparvus* and *An. daciae* populations. Differences in the chromosomal composition of *An. daciae* populations in Crimea and on the Black Sea coast of the Caucasus were revealed. The Crimean population had a low level of polymorphism in autosomal inversions. The data obtained in this study can be used to inform a better control of potential malaria vectors in the Black Sea coastal region.

## Simple Summary

The territories of Crimea and the Black Sea coast of the Caucasus are considered high-risk areas for the resurgence of malaria transmission. Cases of imported malaria are reported annually in these regions. Mosquito species of the genus *Anopheles* (Diptera, Culicidae) inhabit the Black Sea coast, and they can be effective vectors of malaria. The malaria mosquito’s habitat in this region has been affected by the development of tourist resorts over the last 20 years. To understand the current species abundance and distribution, we studied malaria mosquito breeding sites, measured the ecological parameters of water bodies, and determined larval density and species composition of mosquitoes. Seven species of malaria mosquitoes were found in the study areas: *An. atroparvus*, *An. claviger*, *An. daciae*, *An. hyrcanus*, *An. maculipennis*, *An. plumbeus*, and *An. melanoon*. Although the dominant species was *An. maculipennis*, *An. plumbeus* has recently spread to urbanized areas. The level of chromosomal polymorphism in *An. daciae* was significantly higher in the Caucasian populations than in the Crimean population. Data on the species composition and distribution of malaria mosquitoes will help in developing appropriate measures to prevent and control imported malaria and other mosquito-borne diseases.

## 1. Introduction

The environmental and ecological conditions of the Crimean Peninsula and the Black Sea coast of the Caucasus have changed dramatically over the last 20 years [1,2]. Human population growth, the expansion of economic activities, and increased anthropogenic pressure from resort development have led to the destruction and further degradation of natural ecosystems and landscapes and massive environmental pollution. In addition, global warming has an increasingly strong impact on ecosystems, thus constantly changing the epidemiologic situation in the world, including that on the Black Sea coast [3,4]. Although the implementation of antimalarial interventions led to the elimination of autochthonous malaria in Europe [5,6], the territories of Crimea and the Black Sea coast of the Caucasus are currently classified as high-risk areas for the resurgence of malaria transmission [7]. Cases of imported malaria are reported annually in the Crimea and Krasnodar Krai [8,9,10]. Therefore, understanding the current state of epidemiologically important groups of insects, such as malaria mosquitoes of the genus *Anopheles* (Diptera, Culicidae), has become urgent.

Most of the data on species composition and geographical distribution of malaria mosquitoes in the Southern European part of Russia were collected between 1920 and 1960 [11] and are largely out of date. Moreover, the taxonomic status of mosquitoes in the *Maculipennis* complex has changed. Originally, the sibling species of this complex were considered as subspecies of the broadly polytypic species *An. maculipennis* s. l. [12]. Hybridization experiments and numerous data on morphology, ecology, and cytogenetics supported the taxonomic status of the following species of the *Maculipennis* group: *An. atroparvus* Van Theil, 1927; *An. maculipennis* Meigen, 1818; *An. messeae* Falleroni, 1926; *An. melanoon* Hackett, 1934; *An. sacharovi* Favre, 1903; *An. subalpinus* Hackett and Lewis, 1937 [13,14,15,16,17,18]. Later, several sibling species of the *Maculipennis* group were diagnosed using cytogenetic analysis [13,19,20,21,22,23]. Two new species of Palearctic malaria mosquitoes have been distinguished by differences in the banding patterns of the polytene chromosomes: *An. beklemishevi* Stegniy and Kabanova, 1976 [24] and *An. martinius* Shingarev, 1926 [25]. 

Molecular genetic methods provided additional opportunities to discover new malaria mosquito species and became an important approach in determining their taxonomic status [26,27,28]. The most valuable data for *Anopheles* systematics have been obtained by studying the nucleotide composition of the second internal transcribed spacer (*ITS2*) of the ribosomal gene cluster that separates the 5.8S and 28S genes of ribosomal RNA [29]. Three new species have been identified in the *Maculipennis* complex based on the *ITS2* sequence: *An. persiensis* Linton, Sedaghat and Harbach, 2003 [30,31]; *An. daciae* Linton, Nicolescu and Harbach, 2004 [32]; and *An. artemievi* Gordeev, Zvantsov, Goryacheva, Shaikevich and Yezhov, 2005 [33]. *Anopheles subalpinus* has been synonymized with *An. melanoon* because the *ITS2* sequences were identical in both species [34]. In addition, the *ITS2* region of ribosomal DNA served as a tool for reconstructing the phylogenetic relationships in malaria mosquitoes [26,27,35,36,37,38]. Further advances in species recognition and phylogeny reconstruction in the *Maculipennis* group of malaria mosquitoes have been achieved through whole-genome sequencing [39,40].

The geographical distribution and breeding sites of malaria mosquitoes on the Black Sea coast of the Caucasus are poorly studied. Most of the data on species composition were obtained from studies of adults. Mosquito breeding sites of particular cryptic species of the *Maculipennis* group were not determined. According to 2003 and 2008 studies, the list of malaria mosquitoes of the North Caucasus and the Southern Russian Plain included 10 species [41,42,43], nine of which belong to the subgenus *Anopheles*: *An. algeriensis* Theobald, 1903; *An. atroparvus*; *An. claviger* Meigen, 1804; *An. hyrcanus* Pallas, 1771; *An. maculipennis*; *An. melanoon (subalpinus)*; *An. messeae*; *An. plumbeus* Stephens, 1828; and *An. sacharovi*. One species was a member of the subgenus Cellia—*An. superpictus* Grassi, 1899. However, the present geographical distribution of malaria mosquitoes on the Black Sea Coast of the Caucasus and in Crimea is poorly understood.

*Anopheles superpictus* mosquitoes were last found in the Sochi area in 1938 [44]. The rare species *An. algeriensis* was found on the territory of Adygea in 1942 only [45]. A separate study was devoted to the landscape habitat of malaria mosquitoes in the Stavropol Krai [46]. *Anopheles maculipennis* dominates the whole territory of the province. *Anopheles claviger* inhabits the foothills and occurs together with *An. maculipennis* and *An. messeae* s. l. in forest and forest–steppe landscapes. *Anopheles messeae* s. l. is the main species on the steppes, while *An. hyrcanus* dominates in semi-deserts. The main breeding grounds of malaria mosquitoes in Stavropol Krai are reservoirs, ponds, lakes, streams, marshy areas in river floodplains, old rivers, spring mosses, and developed irrigation networks [46]. Cytogenetic studies of malaria mosquitoes were conducted in the south of the Russian Plain, including several locations in Krasnodar Krai and Stavropol Krai. A high level of inversion polymorphism of the sex chromosome and the autosomes 3R and 3L was found in southern populations of *An. messeae* s. l. [47,48]. The relationship between inversion polymorphism and landscape zones on the Black Sea coast of the Caucasus has not been studied.

The available data on the species composition, biology, and ecology of malaria mosquitoes in the Crimean Peninsula are also outdated. The construction and operation of the North Crimean Canal and irrigation systems led to an increase in the breeding places and abundance of malaria mosquitoes in the late 20th and early 21st centuries. Malaria mosquitoes have become indispensable elements of the entomological fauna of agricultural landscapes in lowland Crimea [49]. The development of resort areas resulted in the widespread distribution of blood-sucking mosquitoes, including *Anopheles* mosquitoes, on the territory of the southern coast of Crimea. The lengthening of the period of active mosquito development was observed in resort areas, starting from the first days of March until the end of November [50].

According to the literature data, six species of *Anopheles* were known in Crimea: *An. atroparvus*, *An. claviger*, *An. hyrcanus*, *An. maculipennis*, *An. messeae* s. l., and *An. plumbeus*. The geographic distribution of malaria mosquitoes in the Crimean Peninsula depends on the diversity of the landscapes and breeding sites [51]. *Anopheles claviger* and *An. plumbeus* are common in the mountainous forest zone [50]. *Anopheles claviger* was recorded on the south coast of Crimea and the Chatyr-Dag Plateau, as well as in the Salgir River basin [50,52,53]. *Anopheles atroparvus* inhabits the steppe zone in irrigated areas in the vicinity of Lake Sivash in Northern Crimea [53,54]. *Anopheles hyrcanus* is a rare species and inhabits areas transformed by humans, mainly in the steppe zone [50]. The larval habitats of *An. hyrcanus* in Crimea have not been investigated. *Anopheles maculipennis* is distributed both in the mountainous forest zone and on the steppe plain [50]. It is assumed that the abundance of this species has increased in economically developed areas. The malaria mosquito *An. messeae* s. l. was detected in Crimea based on the analysis of the exochorion pattern of eggs [50,54]. The distribution of *An. messeae* s. l. on the Crimean Peninsula is under discussion. *Anopheles messeae* s. l. was diagnosed by PCR-RFLP in the steppe zone, in the vicinity of Dzhankoy [51]. Separation of the cryptic species *An. daciae* and *An. messeae* s. s. was not performed.

The aim of this work was to revisit and describe in detail the species composition, geographic distribution, breeding sites, and chromosomal polymorphism of the malaria mosquitoes of the Crimean Peninsula and the Black Sea coast of the Caucasus, focusing on the sibling species of the *Maculipennis* group. The dynamics of the epidemic situation under the conditions of global warming and urbanization in the study area were discussed.

## 2. Materials and Methods

### 2.1. Field Collection and Material Preservation

Malaria mosquito larvae were collected from 2009 to 2024 at 56 locations along the Black Sea coast of the Caucasus and the Crimean Peninsula. A total of 2229 individual mosquitoes were included in this study. The coordinates of the collection sites and the number of mosquitoes collected are shown in Table 1. Fourth-instar *Anopheles* larvae were collected from the surface of the water by the dipping method [55] and then were placed in Clark’s fixative solution (glacial acetic acid and 95% ethanol in a 1:3 ratio). Each larva was removed from the Clark’s solution and was divided into two parts. The head and thorax were placed back into the Clark’s solution for inversion polymorphism analysis by cytogenetic methods. The abdomen was fixed in 70% ethanol for molecular identification. All samples were placed in a 1.5 mL Eppendorf tube and stored at −20 °C. The obtained samples were used for preliminary species identification based on morphological characteristics [56,57]. Cryptic species were identified by cytogenetic and molecular genetic markers.

The ecological characteristics of malaria mosquito breeding sites were determined in local habitats. Water temperature (T), potential of hydrogen (pH), and total salinity (ppt) were measured using a Hanna Combo HI 98129 (Hanna Instruments, Woonsocket, RI, USA) conductometer. The dissolved oxygen content in water was measured using an ExStik DO600 oximeter (Extech Instruments, Waltham, MA, USA). Measurements of the environmental parameters of the water were made directly in the water body during larval collection. The device was immersed in water to a depth of 5 centimeters in locations with high larval abundance. The density of larvae of 1–4 stages (number of individuals per m^2^) was estimated in natural habitats (ponds, lakes, and river outfalls) (Table 2). Density counting occurred simultaneously with larval collection. The top layer of water was scooped 10 times with a 25 × 20 × 4 centimeter flat white cuvette. Each scoop contained approximately 50 square centimeters of water surface area. The number of larvae caught was counted per 1 square meter.

### 2.2. Karyotyping

Polytene chromosome preparations were obtained from the salivary glands of fourth-instar larvae according to the standard technique [23,58]. Salivary glands were extracted from the larval thorax with dissecting needles in Clark’s solution. The glands were stained with lacto-aceto-orcein (2% orcein in 80% lactic acid and 100% acetic acid in a 1:1 ratio). Lacto-aceto-orcein staining time was increased from 40 to 80–90 min for best staining. The stained glands were squashed under a coverslip in 50% acetic acid for 15–20 min. Mosquito karyotypes were analyzed using an Eclipse E200 light microscope (Nikon, BioVitrum, Moscow, Russia). The karyotypes were used to identify the sibling species of malaria mosquitoes. Chromosomal inversions were determined by comparing the banding patterns with cytogenetic maps of the studied species [59,60]. Differences in inversion frequencies between mosquito populations were assessed using the Chi-square (χ^2^) test [61]. A total of 2229 individual mosquitoes were karyotyped, of which 75 were *An. atroparvus*, 158 *An. claviger*, 27 *An. hyrcanus*, 908 *An. maculipennis*, 750 *An. daciae*, 309 *An. plumbeus*, and 2 *An. melanoon* larvae.

### 2.3. Genotyping by RLFP-RCR

Karyotyped mosquitoes were used for molecular identification of the sibling species *An. daciae*/*An. messeae*. All samples were analyzed individually. Total DNA was extracted from alcohol-fixed larval fragments (abdomen) using the phenol–chloroform method [62]. The DNA concentration was determined spectrophotometrically using an Implen NanoPhotometer NP80 (Implen, Munich, Germany). The concentration was adjusted to 30–60 ng/µL. Polymerase chain reaction (PCR) was performed at a final volume of 20 μL using an EncycloPlus PCR kit (Eurogen, Moscow, Russia) according to the manufacturer’s instructions.

The species *An. daciae* and *An. messeae* were distinguished by the *ITS2* fragment of the rDNA. The *ITS2* fragment was amplified with the forward primer its2_vdir: 5′-TGTGAACTGCAGGACACATG-3′, and reverse primer its2_nrev: 5′-ATGCTTAAATTTAGGGGGTA-3′, as described previously [60]. RsaI endonuclease (SibEnzyme, Novosibirsk, Russia) was used for PCR restriction fragment length polymorphism (RFLP) analysis [60]. The PCR product *ITS2* in *An. daciae* has 3 restriction sites for RsaI. The lengths of the restriction fragments are 10, 47, 71, and 307 bp. The PCR product *ITS2* in *An. messeae* has 4 restriction sites. The lengths of the restriction fragments are 10, 47, 71, 72, and 235 bp. The obtained PCR products were stained with ethidium bromide and analyzed by electrophoresis in 1.5% agarose gel and TBE (Tris–Borate–EDTA) buffer.

### 2.4. Genotyping by Sequencing

The rDNA-ITS2s of mosquitoes from two Crimean and two Caucasian populations were sequenced. Each larval abdomen was homogenized separately in liquid nitrogen. Genomic DNA was extracted using a standard protocol from the Qiagen DNeasy Blood and Tissue Kit (Qiagen, Germantown, MD, USA). DNA elution was performed in 100 μL of water. *ITS2* from *An. maculipennis* s. l., which do not differ significantly in the electrophoretic mobility of the PCR products (*An. messeae*, *An. daciae*, *An. atroparvus*, and *An. maculipennis*), was amplified with forward universal primers its2_ndir 5′-ATCACTCGGCTCGTGGATCG-3′ or its2_vdir 5′-TGTGAACTGCAGGACACATG-3′ and the reverse primer its2_nrev 5′-ATGCTTAAATTTAGGGGGTA-3′ or its2_rev 5′-ATGCTTAAATTTAGGGGGTAGTC-3′, with modifications [63,64]. The HotStarTaq Plus Master Mix Kit (Qiagen, Germantown, MD, USA) was used for PCR amplification. The PCR mix consisted of a total volume of 20 µL of ~40 ng DNA, 0.5 µM of each forward and reverse primer, and 10 µL of 2× HotStarTaq Plus reaction mix. PCR was performed with the thermocycler Applied Biosystems GeneAmp PCR system 2700 (Applied Biosystems, Waltham, MA, USA) under the following conditions: initial denaturation at 95 °C for 5 min, followed by 25–35 cycles of 95 °C for 15 s, 58 °C for 30 s, and 72 °C for 30 s, and a final elongation step at 72 °C for 5 min. The resulting reaction mix was placed in stand-by mode at 4 °C. The amplicons were visualized by gel electrophoresis in a 2% agarose gel. The DNA amplicons were purified using the Wizard™ PCR Clean Up Kit (Promega, Fitchburg, WI, USA). PCR products were sequenced by Sanger using the BigDye Terminator v3.1 Cycle Sequencing Kit (Thermo Fisher Scientific, Waltham, MA, USA) from suitable forward or reverse primers. The DNA products of the sequencing reactions have been purified by ethanol precipitation and analyzed at the Genomics Core Facility of the Siberian Branch of the Russian Academy of Sciences (http://sequest.niboch.nsc.ru, accessed on 9 March 2022). Nucleotide positions in the *ITS2* sequences of *An. daciae* and *An. messeae* were compared with the reference sequence AY648982 in *An. messeae* [39]. Only the nucleotide ACs in positions 412 and 432 were considered species-specific for *An. daciae*, respectively [39].

*Anopheles melanoon*/*An. maculipennis* s. s. mosquitoes were identified by the BOLD fragment of the mitochondrial *COI* gene using the following Folmer primers: 5′-TTTCAACAAACCATAAGGATATTGG-3′ and 5′-TATACTTCAGGATGACCAAAAAATCA-3′, which were adapted using the “Primer3” program [65]. Total DNA was extracted individually from alcohol-fixed larval fragments (abdomen) using the phenol–chloroform method [62]. PCR amplification was performed at an annealing temperature of 59 °C. PCR was performed in a final volume of 20 μL using an EncycloPlus PCR kit (Eurogen, Moscow, Russia). Elution of fragments from the gel was performed using a Zymoclean™ Gel DNA Recovery Kit (Zymo Research, Los Angeles, CA, USA). The obtained fragments were sequenced by Sanger sequencing. The nucleotide sequence of the PCR fragments was determined from forward and reverse primers on a 3500 Genetic Analyzer using BigDye^®^Terminator v3.1 Cycle Sequencing Kit (Applied Biosystems, Waltham, MA, USA).

### 2.5. Map Production

A map of the geographic location of the sample collection was constructed. The primary construction was carried out in the geoinformation system Panorama 14.0.1 [66]. The final version of the figure was designed in the Adobe Illustrator 2020 program [67].

### 2.6. Bioinformatic and Statistical Analysis

Bioinformatic analysis of the chromatograms was performed using the CromasPro 13.3 software (Technelysium, South Brisbane, Australia) [68]. The sequences obtained by sequencing were aligned to the sequences deposited in the GenBank database using NCBI resources [69]. Statistical analysis of the chromosomal inversion frequencies was performed by Pearson’s Chi-square test using the StatSoft STATISTICA 12.5.192.7 package [70].

## 3. Results

### 3.1. Species Composition, Geographical Distribution, and Ecological Preferences

**Figure 1:**
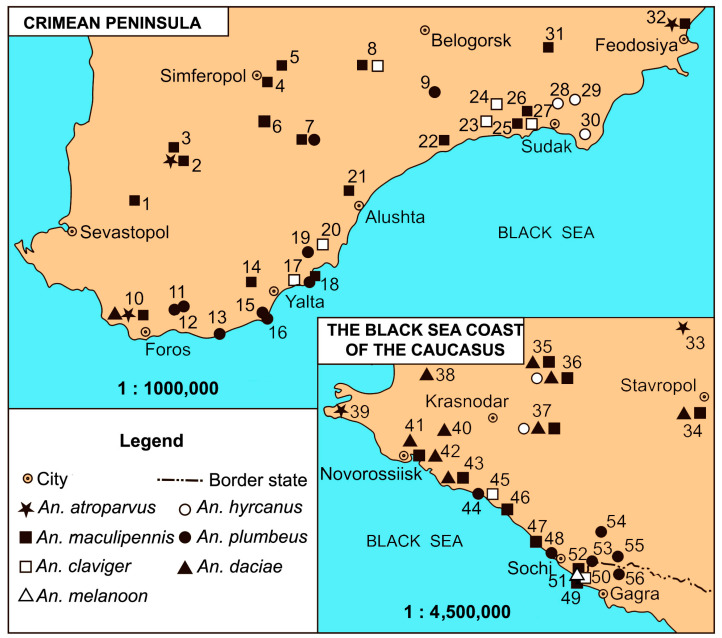
Geographic distribution of malaria mosquitoes on the Crimean Peninsula and the Black Sea coast of the Caucasus. Numbers indicate locations as in Table 1.

Our study found seven species of malaria mosquitoes in the Crimea and the Black Sea coast of the Caucasus: *An. atroparvus*; *An. claviger*; *An. daciae*; *An. hyrcanus*; *An. maculipennis*; *An. melanoon*; and *An. plumbeus* (Table 1). Four of them were sibling species of the Maculipennis group: *An. atroparvus*, *An. daciae*, *An. maculipennis* s. s., and *An. melanoon*. The geographical distribution of the species is shown in Figure 1. Six of them were present in both regions. One species, *An. melanoon*, was found only in the Imereti Valley on the Black Sea coast of the Caucasus (Table 1, location number 51 in the vicinity of Sochi).

*Anopheles maculipennis* s. s. mosquitoes dominated everywhere along the Black Sea coast and in the mountainous forest zone of the Crimean Peninsula (covers almost the entire southern coast of Crimea, from Sevastopol in the west to Feodosia in the east), as well as in the foothills of the Northern Caucasus (on the coast from Sochi to Novorossiysk and at the foot of the Caucasus ridges from Novorossiysk to Krasnodar and Stavropol). This species was found in 28 out of 56 locations (50%). *Anopheles maculipennis* s. s. larvae were found in the most typical *Anopheles* breeding sites, and mosquitoes of this species alone were found in 14 locations (Table 1). Immature stages of *Anopheles maculipennis* s. s. developed in ponds, water reservoirs, drainage and irrigation canals, and in temporary pools of water. Larval development occurred in habitats with a pH range from 6.48 to 8.85. The majority of habitats had pH 7.0–8.4 (Table 2). The total dissolved solids (TDS) in the water in the larval habitats ranged from 0.15 to 2.56 ppt, rarely exceeding 1.5. The average TDS value was 0.76. The larvae are sensitive to dissolved oxygen in the water. The oxygen content varied in the range of 4.4–10.2 mg/L in most breeding sites. The density of *An. maculipennis* s. s. larvae was low at oxygen levels of 0.5–2.3 mg/L. Temperatures in the water bodies where larvae were caught ranged from 16.5 to 33 °C, but the highest density of 160 larvae per square meter was observed at a water temperature of 20.7 °C and an oxygen level of 4.5 mg/L (Table 2; location 3).

*Anopheles daciae* was the most abundant species in the plains located north of the Greater Caucasus Range. *Anopheles daciae* mosquitoes were recorded on the Black Sea coast of the Caucasus near Novorossiysk and Gelendzhik (Figure 1; locations 41–43). A single population of *An. daciae* in Crimea was found near Sevastopol (Figure 1; location 10). The larvae of *An. daciae* developed separately (Table 1; locations 40, 41) or together with *An. maculipennis* s. s. (Table 1; locations 10, 34–37, 42, 43). At some breeding sites shared with *An. maculipennis* s. s., *An. daciae* larvae dominated. At *An. daciae* breeding sites, the water pH ranged from 7.0 to 8.4; the TDS varied between 0.20 and 1.58 ppt; the dissolved oxygen content varied between 0.5 and 8.1 mg/L; and the water temperature ranged from 20.5 to 33.3 °C (Table 2).

*Anopheles atroparvus* mosquitoes were found in steppe biotopes in the Crimea and the North Caucasus, on the Taman Peninsula and the plain in Stavropol Krai (Figure 1; locations 33, 39). Only this species’ larvae were able to develop in highly saline water reservoirs with a TDS of 5.99 ppt (Table 2; location 33). *Anopheles atroparvus* larvae were found together with *An. maculipennis* s. s. and *An. daciae* mosquitoes in ponds and lakes with less saline water with a TDS range of 0.59–1.46 ppt (Table 2; locations 10, 32). The pH value at the *An. atroparvus* breeding sites ranged from 7.14 to 9.1. The daytime water temperature ranged from 18.0 to 28.0 °C, and dissolved oxygen content ranged from 7.7 to 16.5 mg/L.

We found a single breeding site of *An. melanoon* mosquitoes in the Imereti Valley near Sochi (Figure 1; location 51). Two larvae were caught in a temporary water reservoir, such as a stream overflow. *Anopheles melanoon* larvae were identified by the BOLD fragment of the mitochondrial *COI* gene. *Anopheles melanoon* larvae developed together with *An. maculipennis* s. s. in permanent water reservoirs with abundant aquatic vegetation in the floodplain of the Psou River. Many natural breeding sites in the Psou River estuary are now lost due to large-scale construction. *Anopheles maculipennis* s. s. and *An. plumbeus* mosquitoes numerically outnumber *An. melanoon* in the Imereti Valley and neighboring Abkhazia (locations 47–49, 52–56).

*Anopheles hyrcanus* breeding sites were found in lakes along the eastern zone of the Southern Crimean coast. Larvae developed in dense sedge and reed thickets, perennial grasses of the Cyperaceae and Poaceae families, where other malaria mosquito species were absent (Figure 1; locations 28–30). *An. hyrcanus* mosquitoes were found together with *An. maculipennis* s. s. and *An. daciae* larvae in two biotopes in the south of the Azov–Kuban Plain (Figure 1; locations 36–37). The water pH ranged from 7.00 to 8.24 in the *An. hyrcanus* breeding places. The daytime temperature varies between 22.4 and 33.3 °C. The TDS changed from 0.81 to 3.02 ppt, and the dissolved oxygen content in the water reached 10.0 mg/L (Table 2).

Because *Anopheles claviger* is a highly specialized malaria mosquito species, its larvae were found in springs, ditches with running water, or in water reservoirs fed by groundwater. The water temperatures ranged from 16.5 to 23.7 °C at most of the breeding sites (Table 2). Higher water temperatures of up to 27.5 °C were only recorded in one biotope, with flowing water and a low density of *An. claviger* larvae (Table 2; location 50). The water pH varied within a narrow range of 7.14–7.72, but it was 9.26 in one biotope (Table 2; location 50). The TDS in all locations varied between 0.24 and 2.08 ppt. The amount of dissolved oxygen in the water was 2.5 and 7.6 mg/L (Table 2; locations 45 and 50).

*Anopheles plumbeus* mosquitoes were found in the mountainous forested part of the Southern Crimean coast. Larvae developed in shallow forest lakes, the overflow of small rivers, in temporary micro-habitats such as tree holes, and condensation puddles between piles of stones (Table 1; locations 7, 9, 11–13, 15, 16, 18, 19). Breeding sites of this species were found in tree holes on the Black Sea coast of the Caucasus (locations 48, 53) and in mountains at altitudes up to 1700 m (Table 1; locations 54, 55). Breeding sites of *An. plumbeus* mosquitoes were found in old car tires in Caucasian resorts (Table 1; locations 44, 53, 56). The water composition in the larval habitats was highly variable (Table 2). Water acidity correlated with organic matter content, and the pH varied from 5.20 to 8.32 (in open biotopes, mainly in the range from 7.14 to 7.62). The TDS varied between 0.03 and 2.78 ppt. The daytime water temperatures at breeding sites ranged from 14.9 to 32.3 °C. The dissolved oxygen content was measured in a number of micro-watersheds and ranged from 2.5 to 7.5 mg/L. The larval densities did not exceed nine per square meter at most breeding sites, but in habitats with high saprobic conditions, larval densities reached 32–90 mosquitoes of all instars per square meter. *Anopheles plumbeus* larvae developed together with *An. maculipennis* s. s. in the two largest of the listed breeding sites—in a forest lake and in an ornamental pond of the Nikitsky Botanical Gardens (Table 1; locations 7, 18).

### 3.2. Chromosomal Inversion Polymorphism

Chromosomal inversions were studied in populations of *An. atroparvus* and *An. daciae*. We identified chromosomal inversions in accordance with the previously published photo maps of the polytene chromosomes in *An. atroparvus* and *An. daciae/An. messeae* [59,60].

*Anopheles daciae* differed from other species by a high degree of chromosomal polymorphism (Table 3).

**Figure 2:**
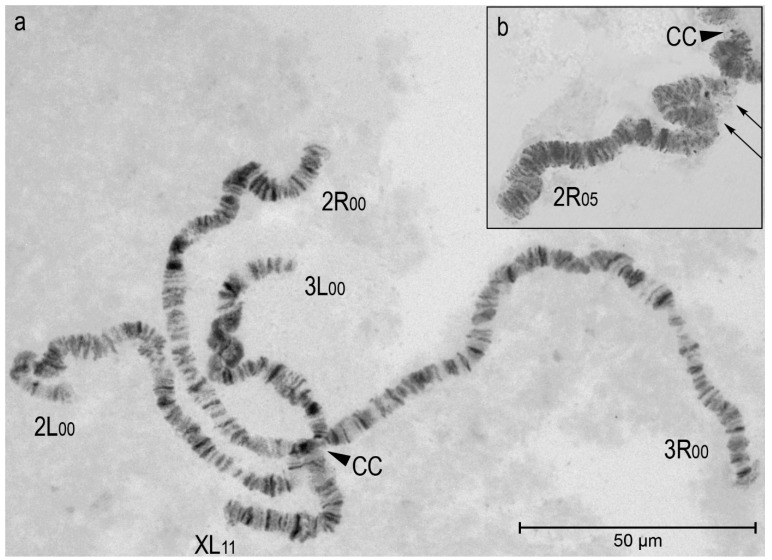
A chromosomal complement of a squashed preparation of salivary gland cells in an *An. daciae* female, stained by lacto-aceto-orcein. Panel (**a**) shows the standard karyotype XL_11_, 2R_00_, 2L_00_, 3R_00_, and 3L_00_, where XL, 2R, 2L, 3R, and 3L represent chromosome arms and the numbers _11_ and _00_ are chromosomal variants (objective lens—Nikon Plan Fluor 60×/0.85). Chromosome arms XL, 2R, 2L, 3R, and 3L are indicated. Panel (**b**) shows the inversion heterozygote 2R_05_ (11c-14a) in *An. daciae* (objective lens—Nikon Plan 100×/1.25). The arrows indicate the points of breaks and homologous exchanges in the inversion loop. CC stands for the chromocenter. Scale bar equals 50 μm.

Homo- and heterozygotes for three paracentric inversions were common in populations of this species: XL_1_ (2a-5b); 3R_1_ (23c/24a-26c/27a); and 3L_1_ (34b/c-37a/b-38c/39a-39c/d), whereas the 3L_1_ inversion consists of two overlapping inversions. Only one heterozygote 2R_05_ for new inversion 2R_5_ (11c-14a) was found in the Novorossiysk population (Figure 2; location 42). There were no inversions in the 2L arm.

**Figure 3:**
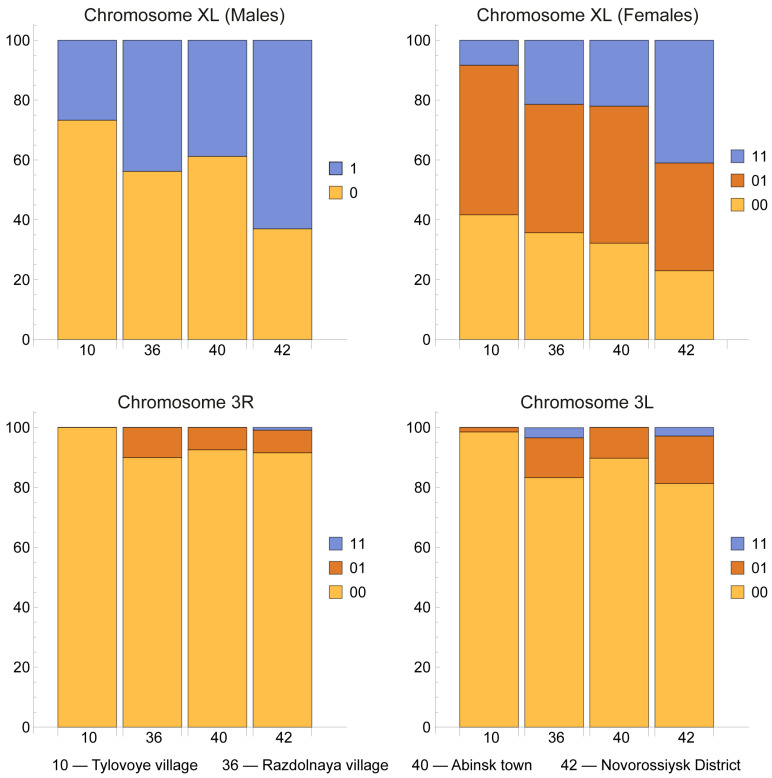
Frequencies of inversion homo- and heterozygotes in populations of *Anopheles daciae* in Crimea (location 10) and on the Black Sea coast of the Caucasus (locations 36, 40, and 42). The *X*-axis shows the numbers of locations; the *Y*-axis shows the frequencies of homo- and heterozygotes, in %. The frequencies of inversions of sex chromosome XL in males and females are given separately. The frequencies of inversions of 3R and 3L autosomes are shown for individuals of both sexes.

We described, for the first time, the chromosomal polymorphism of marginal populations of *An. daciae* in the southern part of the species range. Only one population of *An. daciae* in the Crimean Peninsula (Figure 3; location 10) differed in chromosomal composition from three Caucasian populations (Figure 3; locations 36, 40, 42).

A high level of inversion polymorphism of the sex chromosome XL was observed in all populations of *An. daciae* on the Black Sea coast (Figure 3). The frequency of XL_0_ inversions was significantly higher in males in the Crimean Peninsula population compared to males from the Caucasian populations (χ^2^ = 4.47; number of degrees of freedom df = 1; *p* < 0.05). The frequency of the homo- and heterozygotes XL_00_ and XL_01_ in females from the Crimean Peninsula population was higher than in females from the Caucasian populations (χ^2^ = 6.16; df = 2; *p* < 0.05).

The autosomal inversions 3R_1_ and 3L_1_ are present with low frequency in coastal populations of *An. daciae* (Figure 3). The frequencies of homo- and heterozygotes with 3R_1_ and 3L_1_ inversions were significantly higher in the Caucasian populations (χ^2^ = 4.48 and 7.40; df = 1; *p* < 0.05 and *p* < 0.01, respectively). In general, the level of chromosomal polymorphism was significantly higher in the Caucasian populations than in the Crimean population.

**Figure 4:**
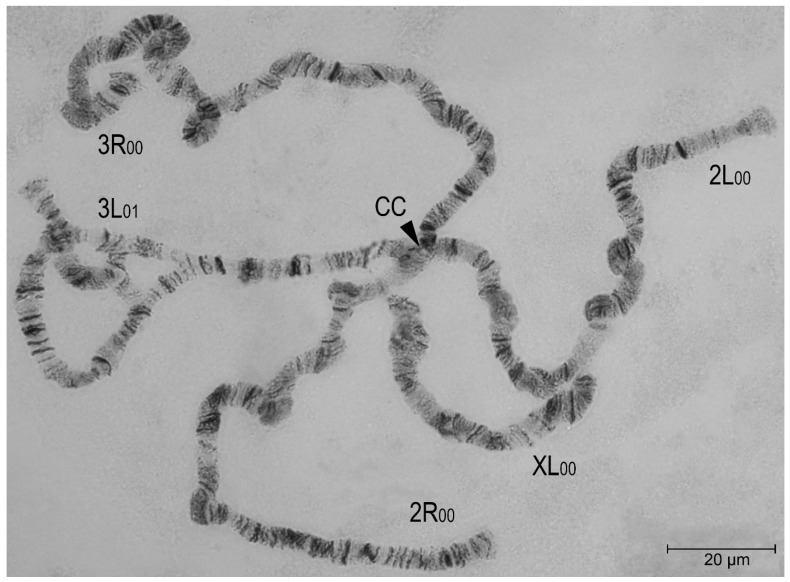
A chromosomal complement of a squashed preparation of salivary gland cells in an *Anopheles atroparvus* female, with karyotype XL_00_, 2R_00_, 2L_00_, 3R_00_, and 3L_01_, stained by lacto-aceto-orcein, where XL, 2R, 2L, 3R, and 3L represent chromosome arms and the numbers _00_ and _01_ are chromosomal variants (objective lens—Nikon Plan 100×/1.25). The inversion heterozygous variant 3L_01_ (34b/c-38b) in *An. atroparvus* appears as a loop in the chromosome. CC stands for the chromocenter. Chromosome arms XL, 2R, 2L, 3R, and 3L are indicated. Scale bar equals 20 μm.

The inversion polymorphism was observed in *An. atroparvus* populations. Heterozygotes for inversion 3L_1_ (34b/c-38b) were found in two populations from Crimea and the Stavropol Region (Figure 4).

The heterozygotes 3L_1_ occurred with a frequency of 33.3% in both populations (Table 1; locations 2, 19). No homozygotes for this inversion were detected. Chromosome 3L has an identical disc pattern in *An. atroparvus* and *An. daciae*. Interestingly, the breakpoints of the 3L_1_ inversion of *An. atroparvus* (34b/c and 38b) are close to two breakpoints of the overlapping 3L_1_ inversion of *An. daciae* (34b/c-38c/39a). Individuals of both species with these inversions occur in the same regions.

## 4. Discussion

In this study, we studied the species composition, geographical distribution, and physicochemical properties of malaria mosquito breeding places on the Crimean Peninsula and the Black Sea coast of the Caucasus. *Anopheles maculipennis* s. s. was the most abundant species in the foothills and coastal areas. *Anopheles daciae* dominated in freshwaters in the Kuban–Priazov lowland. *Anopheles atroparvus* larvae developed in saline lakes, but they may also be subdominant in freshwater pools. *Anopheles melanoon* mosquitoes were found only in the humid subtropical zone of the Black Sea coast of the Caucasus. *Anopheles claviger* mosquitoes preferred cool springs and groundwater outflows. *Anopheles hyrcanus* larvae developed in reed thickets along the shores of shallow lakes. *Anopheles plumbeus* larvae inhabited tree holes and shallow temporary water pools. The diversity of the coastal landscapes allowed the above species with different ecological preferences to co-occur in the same area. The number of malaria mosquitoes was limited by the lack of suitable water pools during dry and hot summers, especially in the steppe zone.

*Anopheles maculipennis* s. s. numerically outnumbers the mosquitoes of other species of the *Maculipennis* complex in water pools with standing fresh water on the southern coast of the Crimea and in the foothills of the Caucasus. *Anopheles maculipennis* larvae develop together with *An. daciae* in Krasnodar and Stavropol Krai. Joint development of *An. maculipennis*, *An. daciae*, and *An. messeae* s. s. is also shown in the center and north of the Russian Plain [40,71]. Chromosomal polymorphism in *An. maculipennis* populations has been noticed, but it was not evaluated in the current study. Populations of this species are considered chromosomally monomorphic throughout the territory of the Russian Plain. Only one single heterozygous inversion on the left arm of chromosome 2L, at the 5c-20c region, was observed in the population of Falesti [59,72].

Before the description of *An. daciae* in 2004 [32], both *An. daciae* and *An. messeae* were diagnosed as *An. messeae* [11,41,53,54]. In other studies using molecular genetic methods, *An. messeae* s. s. was not found in the south of European Russia. The southern boundary of the range of *An. messeae* s. s. was found to the north in the forest zone of the Russian Plain [63]. We were unable to find any *An. messeae* s. s. mosquitoes in the studied regions. This result was verified by sequencing the *ITS2* fragments of 166 larvae from three habitats (Table 1; locations 10, 40, 42) [63]. The ecological niches of *An. daciae* and *An. maculipennis* partially overlap in the Russian Plain; larvae of both species can develop in the same water reservoirs under similar temperate conditions. However, more suitable breeding sites for *An. maculipennis* were found in the subtropical zone on the southern coast of the Crimea and in the southern part of the Black Sea coast of the Caucasus, where *Anopheles daciae* mosquitoes were not found. The southern range limit of *An. daciae* was in the Pshadsky district of Krasnodar Krai (Figure 1; location 43), where the transition from a temperate to a subtropical climate occurs.

Winter temperatures are significantly higher in southern areas, in the Tuapse–Sochi coastal strip, making over-wintering conditions the main limiting factor for the Palearctic species of malaria mosquitoes in the south [73]. It is likely that diapausing females of *An. daciae* cannot tolerate warm winters in the humid subtropical zone. In contrast, *An. maculipennis* s. s. females can blood-feed repeatedly during the over-wintering period [7,73,74,75]. Feeding on blood during diapause helps species to survive in excessively warm winter shelters, whereas fat reserves of diapausing females are rapidly depleted.

So far, only *An. maculipennis* s. s. has been considered as the main potential vector of malaria throughout the Southern Russian Plain, especially in the mountainous and foothill areas of the North Caucasus [7]. However, we believe that the epidemiologic role of *An. daciae* is currently underestimated and needs to be further evaluated.

Our study indicated that the populations of *An. daciae* and *An. maculipennis* s. s. on the Crimean Peninsula were isolated from those of the Kuban–Priazov lowland and the Black Sea lowland. A natural barrier for these mosquitoes is the dry steppes with saline lakes in the Northern Crimea and the Taman Peninsula. The isolation of populations likely occurred after the last glaciation in Europe because of the Black Sea natural disaster [76]. The Black Sea was a freshwater lake with a level 120 m below the present-day level. The rupture of the Bosphorus, followed by the intrusion of saline water from the Mediterranean Sea, occurred around 9300 BC. Prior to this, a significant part of the present underwater continental shelf of the Black Sea was a terrestrial area with a common flora and fauna. Flooding led to the breakage of a continuous coastal strip and the formation of the Crimean Peninsula. The subsequent climatic aridification in the northern part of Crimea led to the isolation of the mosquito fauna of the Southern Crimean coast [76]. The use of inversions as genetic markers allows for reconstructing the genetic history of *An. daciae* populations on the Black Sea coast. Apparently, long-term isolation caused differences in the chromosomal composition of *An. daciae* populations in Crimea and on the Black Sea coast of the Caucasus. The Crimean population had a low level of polymorphism in autosomal inversions: 0% homo- and heterozygotes for inversion 3R_1_ and only 1.5% heterozygotes for inversion 3L_1_ (Table 3; location 10). In Caucasian populations, the frequencies of homo- and heterozygotes for 3R_1_ and 3L_1_ inversions were much higher and vary between 7.4–10.0% and between 10.2–18.7%, respectively (Table 3; location 36, 40, 42). We determined that the Crimean and the Black Sea coast populations of this species were more homogeneous than in the center of the Russian Plain. The long-term isolation of the Crimean populations has led to even more pronounced differences in the chromosomal polymorphism of the *An. daciae* populations there. Local populations within Crimea differ mainly in the frequency of inversions in the sex chromosome XL. XL_0_ inversion serves as a species marker of *An. daciae*. The alternative inversion XL_1_ is considered ancestral to the two cryptic species *An. daciae* and *An. messeae* [60]. According to molecular genetic analyses, the separation of *An. daciae* and *An. messeae* happened about 2 Ma during the glaciation of Eurasia [40]. It is assumed that *An. daciae* mosquitoes were isolated in a refugium in Southern Europe. The XL_0_ inversion, which occurs with high frequency in populations of the steppe zone and broad-leaved forest zone, probably arose during this period [77]. The autosomal inversions 3R_1_ and 3L_1_ occur with lower frequency than in the center of the Russian Plain [39]. These inversions were probably derived from the common ancestor of *An. daciae* and *An. messeae*, since the polymorphism on inversions 3R_1_ and 3L_1_ is present in the populations of both species. Geographic gradients in longitude suggest the influence of climatic factors on the frequencies of these inversions [63]. The level of chromosomal polymorphism in *An. daciae/An. messeae* mosquitoes was shown to be associated with specific landscape–climatic zones [77]. We believe that the high level of inversion polymorphism is associated with optimal landscape–climatic zones for this species. The sub-taiga and forest–steppe zones are probably more favorable for the development of *An. daciae* mosquitoes than the steppes of the Kuban–Priazov lowland.

In contrast to *An. daciae* and *An. maculipennis* s. s., *An. atroparvus* mosquitoes develop en masse in the steppe salt lakes in the Northern Crimea [78]. The steppes and semi-deserts of the Northern Crimea belong to the same natural zones as the Black Sea lowlands. The steppes of the Kerch Peninsula in Crimea are separated from the steppes of the Taman Peninsula by the relatively narrow Kerch Strait. Thus, the range of *An. atroparvus* covered the coastal areas as a continuous strip. Similar chromosomal variability occurred in geographically distant populations of this species. For example, the chromosomal inversion 3L_1_ is present in the Crimea and on the Black Sea coast of the Caucasus in this mosquito species. The northern limit of the *An. atroparvus* range is the Southern Russian Plain, approximately south of the 48th parallel [54], but it is not well defined.

The malaria mosquito *An. melanoon* was one of the rarest species of the *Maculipennis* complex on the Black Sea coast of the Caucasus. This species lives in sympatry with *An. maculipennis* s. s. in the territory of Bulgaria, Moldova, Romania, Georgia, and Turkey [32,79,80,81,82,83]. We assumed that the northern limit of the *An. melanoon* distribution was the subtropical zone of the Black Sea coast of the Caucasus and may have shifted southwards to the Imereti Valley under conditions of anthropogenic transformation of coastal landscapes. The species was chromosomally monomorphic [18]. The polytene chromosomes of *An. melanoon* and *An. maculipennis* s. s. in ovarian nurse cells have the same banding patterns, except for the pericentromeric regions [84] that attach to the nuclear periphery, which causes the differentiation of chromosomal morphology between these species [85,86,87].

The malaria mosquito *An. hyrcanus* inhabited the steppe plains and was one of the most highly specialized species. The larvae of this exophilic mosquito developed mainly in the coastal zone of lakes and in wetlands covered with reeds, rushes, sedges, cattails, and other aquatic vegetation. In the Krasnodar Krai, rice fields created favorable breeding conditions for *An. hyrcanus* mosquitoes. *Anopheles hyrcanus* breeding sites have been found in the Tien Shan and Pamir–Alai valleys of Central Asia at altitudes of up to 1000 m [88]. Chromosomal polymorphism in *An. hyrcanus* populations has not been studied. A high level of chromosomal variability was found in populations of another species of the Oriental Hyrcanus group, *An. kleini* Rueda, 2005, which lives in the Far East [89,90].

*Anopheles claviger* mosquitoes were widespread in the foothills of the Caucasus and Crimea. We studied several *An. claviger* breeding sites on the southern coast of Crimea. The natural complexes of the southern coast of Crimea were formed under the conditions of a hot sub-Mediterranean climate [91]. The sub-Mediterranean climate zone consists of a western part (from Cape Aya to Alushta) and a more arid eastern part (from Alushta to Feodosia). *Anopheles claviger* mosquitoes inhabited both parts of the sub-Mediterranean climate zone, especially in mountain forests at the northern limit of this zone. Two population peaks of *An. claviger* mosquitoes have been recorded during the summer season: in mid–late June and late October [47]. The summer decline in abundance was specific for *An. claviger* throughout the Southern Russian Plain. *Anopheles claviger* can be found at altitudes of up to 2200 m in the Tien Shan Mountains and numerically outnumbers other *Anopheles* species in the foothills throughout the breeding season [92]. A sharp decline in *An. claviger* mosquito numbers was shown to occur during the summer months in the lowlands of Central Asia, as reservoirs of cold clear water are required for the larval development of this species. Chromosomal variability in the wide distribution of *An. claviger* has not been studied.

*Anopheles plumbeus* mosquitoes were usually found in the mountainous forests. The larval stages of this species developed in the rotten tree holes filled with rainwater with a high organic content, as well as in small water pools of natural origin [47]. *Anopheles plumbeus* females laid eggs in water pools just above the waterline, and the larvae hatch during the first floods of the rainy season [93]. *Anopheles plumbeus* was originally considered a dendrolimnetic species, but the ecological preferences of this species have now changed [94,95]. In several European countries, this species has begun to breed in water pools of artificial origin, such as rainwater barrels, lagoons, septic tanks, car tires, cemetery vases, liquid manure collection tanks, and manure puddles [94,95,96,97,98]. According to our observations, *An. plumbeus* has started to breed in the territory of Caucasian resorts since 2018. The anthropogenic transformation of coastal landscapes has created favorable conditions for the development of this species in artificial water containers with hard walls polluted with organic matter. An increase in the number of *An. plumbeus* in Crimea and the Black Sea coast of the Caucasus began in 2018. The expansion of the *An. plumbeus* range from the vicinity of Sochi city (Table 1; location 53) to the Tuapse district (Table 1; location 44) occurred in several stages and was similar to the spread of the invasive Asian tiger mosquito *Aedes albopictus* Scuse, 1895 [99]. Unlike other Eurasian malaria mosquito species, *An. plumbeus* has been shown to be capable of transmitting malaria parasites *Plasmodium falciparum* and *Plasmodium vivax* [94,100,101]. This malaria mosquito was considered the probable cause of two autochthonous cases of *Pl. falciparum* malaria in Germany [75,102]. Moreover, *An. plumbeus* was shown to be susceptible to West Nile virus and, because of its double ornithophagic and anthropophagic behavior, was considered one of the potential vectors of this virus from birds to humans [103,104]. Clearly, the epidemiological significance of this species as a secondary vector of vector-borne diseases in Crimea and the Black Sea coast of the Caucasus needs to be reconsidered. It remains unknown how changes in the ecological preferences of *An. plumbeus* are related to the genetic variability of natural populations. The chromosomal polymorphism in *An. plumbeus* populations has not been studied.

Although *An. algeriensis*, *An. sacharovi*, and *An. superpictus* were previously recorded in the Crimea and the Black Sea coast of the Caucasus [54], the breeding places of these species have not been found at the present time. Previously, *An. algeriensis* was found in spring water in the foothills of the North Caucasus and the Kuban–Priazov lowland [105]. *Anopheles algeriensis* larvae begin to emerge in spring waters in the Caucasus at a temperature of about 5 °C [106]. The breeding sites of mosquitoes of this species have not been found less than 3–5 km from any settlement [107]. The most recent record of *An. algeriensis* mosquitoes was made in Kalmykia [108]. The northern limit of the range of *An. algeriensis* is south of the 48th parallel on the Russian Plain and is not well defined. *Anopheles algeriensis* has been shown to be a competent vector for *Plasmodium* parasites [109]. However, this species is rare, lives far from human settlements, and may not play a role in the malaria transmission in the Caucasus. The karyotype composition and chromosomal polymorphism in *An. algeriensis* populations have not been studied.

The malaria mosquito *An. sacharovi* has not been recorded in Crimea and the Black Sea coast of the Caucasus, but it has been found in the adjacent plains of Transcaucasia and Dagestan [110]. This species was the main vector of three-day malaria caused by *Pl. vivax* in the valleys of Transcaucasia (Georgia, Armenia, and Azerbaijan) during the malaria outbreak in the late 20th and early 21st centuries [7]. The malaria mosquitoes *An. sacharovi* and *An. superpictus* are among the most epidemiologically important species in the Palearctic [7]. Global warming may contribute to the expansion of the range of these species to the Southern Russian Plain [111,112,113]. For example, the northern limit of the range of *An. sacharovi* has shifted from Dagestan to the territory of Kalmykia, where this species was not previously recorded [110]. Environmental changes can affect not only the geographical distribution but also the abundance and ecological preferences of malaria mosquitoes. Finally, *An. superpictus* was considered a mountain stream species in Central Asia (Tajikistan). Currently, mosquitoes of this species are found in lowland waters with increased eutrophication, including rice fields. The abundance of *An. superpictus* increased significantly as a result of changes in ecological preferences [88].

## 5. Conclusions

Our findings suggest that malaria mosquito species differ in their geographical distribution and physicochemical characteristics of breeding sites within the Crimean Peninsula and the Black Sea coast of the Caucasus. The list of malaria mosquito species of the studied regions has been updated. The breeding places of seven species of malarial mosquitoes were found in the studied area, namely *An. atroparvus*, *An. claviger*, *An. daciae* (formerly identified as *An. messeae* s. l.), *An. hyrcanus*, *An. maculipennis* s. s., *An. plumbeus*, and *An. melanoon*. *Anopheles maculipennis* was found most frequently at different breeding locations. Urbanization affects the distribution of malaria mosquitoes. *An. plumbeus* is dispersed in urbanized settlements in coastal resort areas of the Caucasus. Chromosomal polymorphism was found in populations of *An. atroparvus* and *An. daciae*. The frequencies of autosomal inversions in the isolated population of *An. daciae* on the Crimean Peninsula were significantly lower compared to the frequencies of these inversions on the Black Sea coast of the Caucasus. Further monitoring of the composition of malaria vector species and their geographic distribution represents an important component of entomological surveillance, which stimulates the development of appropriate mosquito control strategies aimed at preventing the re-emergence and spread of malaria in areas where it was previously eliminated.

## Figures and Tables

**Table 1:** Species composition of malaria mosquitoes in breeding places of the Crimean Peninsula and the Black Sea coast of the Caucasus.

No.	Location/Breeding Habitat Type	Latitude	Longitude	Date of Sampling	Number (%) of Mosquitoes							
					Total	*AT*	*CL*	*HY*	*MA*	*DA*	*PL*	*ML*
	***Crimean Peninsula***											
1	Pirogovka village, Nakhimov district of Sevastopol/ water storage	44.685296	33.739026	11.09.2016	18	-	-	-	18 (100)	-	-	-
2	Bakhchisaray town/pond	44.763889	33.853056	10.09.2016	12	3 (25.0)	-	-	9 (75.0)	-	-	-
3	Bakhchisaray town/ dried-up creek	44.763889	33.853611	10.09.2016	10	-	-	-	10 (100)	-	-	-
4	Simferopol city, botanical garden/pond	44.939167	34.133056	10.09.2016	15	-	-	-	15 (100)	-	-	-
5 *^1^	Mazanka village, Simferopol district/pond	45.014861	34.235861	12.07.2016	57	-	-	-	57 (100)	-	-	-
6	Konstantinovka village, Simferopol district/lake	44.856389	34.123333	20.08.2016	3	-	-	-	3 (100)	-	-	-
7	Mramornoye village, Simferopol district/lake	44.813889	34.237222	20.06.2016	9	-	-	-	7 (77.8)	-	2 (22.2)	-
8	Mezhgorye village, Belogorsky district/river	44.970556	34.416111	20.06.2016	9	-	5 (55.6)	-	4 (44.4)	-	-	-
9	Krasnosyolovka village, Belogorsky district/river spill	44.917778	34.633333	20.06.2016	2	-	-	-	-	-	2 (100)	-
10A	Tylovoye village, Balaklava district of Sevastopol/pond	44.441389	33.728056	13.09.2016	128	2 (1.6)	-	-	60 (46.9)	66 (51.5)	-	-
10B *^2^		44.443570	33.739879	12.08.2017	21	-	-	-	12 (57.1)	9 (42.9)	-	-
10C					84	1 (1.2)	-	-	83 (98.8)	-	-	-
10D *^3^		44.441740	33.727469	08.08.2019	53	-	-	-	23 (43.4)	30 (56.6)	-	-
10E					95	-	-	-	55 (57.9)	40 (42.1)	-	-
11	Rodnikovoye village, Balaklava district of Sevastopol/puddle	44.453611	33.862222	20.07.2016	3	-	-	-	-	-	3 (100)	-
12	Rodnikovoye village, Balaklava district of Sevastopol/tree hollow	44.457222	33.872778	20.07.2016	7	-	-	-	-	-	7 (100)	-
13	Simeiz Settlement, Yalta district/mountain puddle	44.403611	33.991667	20.06.2016	5	-	-	-	-	-	5 (100)	-
14	Yalta district/forest puddle	44.516389	34.143889	20.07.2016	37	-	-	-	37 (100)	-	-	-
15	Gaspra settlement, Yalta district/water in rock cracks	44.433611	34.130000	20.06.2016	3	-	-	-	-	-	3 (100)	-
16	Gaspra settlement, Yalta district/tree hollow	44.445278	34.118889	20.06.2016	2	-	-	-	-	-	2 (100)	-
17	Voskhod settlement, Yalta district/lake	44.517417	34.219796	20.08.2016	8	-	8 (100)	-	-	-	-	-
18	Nikitsky Botanical Gardens, Yalta district/pond	44.508831	34.233093	13.09.2016	7	-	-	-	1 (14.3)	-	6 (85.7)	-
19	Krasnokamenka village, Yalta district/forest puddle	44.577500	34.255556	20.06.2016	2	-	-	-	-	-	2 (100)	-
20	Zaprudnoye village, Alushta district/lake	44.599444	34.305000	20.08.2016	14	-	14 (100)	-	-	-	-	-
21	Nizhnyaya Kutuzovka village, Alushta district/pond	44.709842	34.377544	14.09.2016	31	-	-	-	31 (100)	-	-	-
22	Alushta district/pond	44.814167	34.657778	14.09.2016	12	-	-	-	12 (100)	-	-	-
23	Gromovka village, Sudak district/pond	44.857778	34.791389	20.06.2016	3	-	3 (100)	-	-	-	-	-
24	Voron village, Sudak district/spring	44.892222	34.820278	20.08.2016	3	-	3 (100)	-	-	-	-	-
25	Veseloye village, Sudak district/water reserve	44.849722	34.883611	15.09.2016	9	-	-	-	9 (100)	-	-	-
26	Sudak district/pond	44.868611	34.900556	15.09.2016	3	-	-	-	3 (100)	-	-	-
27	Veseloye village, Sudak district/lake	44.851944	34.883333	20.08.2016	2	-	2 (100)	-	-	-	-	-
28	Dachnoye village, Sudak district/river spill	44.888889	34.990278	20.08.2016	1	-	-	1 (100)	-	-	-	-
29	Dachnoye village, Sudak district/lake	44.897362	35.040173	20.08.2016	4	-	-	4 (100)	-	-	-	-
30	Mindalnoye village, Sudak district/lake	44.831756	35.082243	20.07.2016	3	-	-	3 (100)	-	-	-	-
31	Grushevka village, Sudak district/lake	45.010570	34.971796	15.09.2016	48	-	-	-	48 (100)	-	-	-
32	Feodosia city/pond	45.063792	35.341071	16.09.2016	9	7 (77.8)	-	-	2 (22.2)	-	-	-
	***Black Sea coast of the Caucasus***											
33	Krasnogvardeyskoye village, Stavropol Krai/dried up river	45.850476	41.482381	12.08.2015	27	27 (100)	-	-	-	-	-	-
34	Stavropol city/pond	45.013332	41.974723	12.08.2015	30	-	-	-	26 (86.7)	4 (13.3)	-	-
35	Malevanyi settlement, Krasnodar Krai/river	45.531517	39.461591	21.08.2015	50	-	-	-	1 (2.0)	49 (98.0)	-	-
36	Razdolnaya village, Korenovsky district, Krasnodar Krai/lake	45.383469	39.537257	01.08.2024	100	-	-	18 (18.0)	52 (52.0)	30 (30.0)	-	-
37	Shengzhiy settlement, Republic of Adygeya/channel	44.883810	39.075139	05.08.2009	54	-	-	1 (1.9)	2 (3.7)	51 (94.4)	-	-
38	Novonikolayevskaya village, Krasnodar Krai/pond	45.581165	38.369233	04.08.2019	120	-	-	-	-	120 (100)	-	-
39	Tamanskoye Settlement, Temryuksky district, Krasnodar Krai/lake	45.144803	36.700849	07.07.2016	35	35 (100)	-	-	-	-	-	-
40 *^4^	Abinsk town, Krasnodar Krai/pond	44.862380	38.183556	14.08.2018	108	-	-	-	-	108 (100)	-	-
41	Gaiduk village, Novorossiysk district, Krasnodar Krai/pond	44.781486	37.679653	03.08.2018	100	-	-	-	-	100 (100)	-	-
42	Novorossiysk district, Krasnodar Krai/water storage	44.780000	37.815833	09.07.2016	108	-	-	-	1 (0.9)	107 (99.1)	-	-
43	Pshada village, Gelendzhik district, Krasnodar Krai/river	44.452257	38.346501	19.08.2015	106	-	-	-	70 (66.0)	36 (34.0)	-	-
44	Community Zarya, Tuapse district, Krasnodar Krai/ car tire	44.082778	39.131667	31.07.2021	36	-	-	-	-	-	36 (100)	-
45	Novomikhailovsky settlement, Tuapse district, Krasnodar Krai/drainage ditch	44.247752	38.844524	18.08.2015	100	-	100 (100)	-	-	-	-	-
46	Agui-Shapsug village, Tuapse district, Krasnodar Krai/river	44.174722	39.066944	11.07.2016	35	-	-	-	35 (100)	-	-	-
47	Zubova Shchel village, Sochi district, Krasnodar Krai/river	43.837451	39.441109	13.07.2016	34	-	-	-	34 (100)	-	-	-
48	Sochi city, Krasnodar Krai/tree hollow	43.675833	39.608889	30.07.2021	60	-	-	-	-	-	60 (100)	-
49	Adler town, Krasnodar Krai/ swamp	43.432222	39.947222	17.07.2016	32	-	-	-	32 (100)	-	-	-
50A	Verkhneveseloye village, Sochi district, Krasnodar Krai/drainage ditch	43.426067	39.973288	04.08.2023	14	-	14 (100)	-	-	-	-	-
50B *^5^					6	-	6 (100)	-	-	-	-	-
50C		43.426306	39.973515	04.08.2024	3	-	3 (100)	-	-	-	-	-
51 *^6^	Sochi city, Krasnodar Krai/stream	43.410321	39.983947	07.08.2024	2	-	-	-	-	-	-	2 (100)
52	Sirius settlement, Krasnodar Krai/fire pond	43.412778	39.937778	16.07.2016	156	-	-	-	156 (100)	-	-	-
53	Vesyoloye microdistrict, Sochi city, Krasnodar Krai/car tire	43.409722	40.008330	11.08.2018	32	-	-	-	-	-	32 (100)	-
54	Krasnaya Polyana Resort, Sochi district, Krasnodar Krai/hollow tree	43.711944	40.209167	25.07.2021	94	-	-	-	-	-	94 (100)	-
55	Rosa Khutor resort, Sochi district, Krasnodar Krai/ hollow tree	43.638978	40.307983	29.07.2021	39	-	-	-	-	-	39 (100)	-
56	Ritsinsky National Park, Gudauta district, Abkhazia/ car tire	43.473889	40.538056	24.07.2021	16	-	-	-	-	-	16 (100)	-
Total					2229	75	158	27	908	750	309	2

**No**., location number; **AT**—*An. atroparvus*; **CL**—*An. claviger*; **HY**—*An. hyrcanus*; **MA**—*An. maculipennis*; **DA**—*An. daciae*; **PL**—*An. plumbeus*; **ML**—*An. melanoon*. *^1^—sequences accession numbers GenBank ID PQ510968–PQ511024 (57); *^2^—sequences accession numbers PQ554994–PQ555014 (21); *^3^—sequences accession numbers PQ550752–PQ550804 (53); *^4^—sequences accession numbers PQ526514–PQ526598 (85); *^5^—sequences accession numbers PQ740514–PQ740518 (5); *^6^—sequences accession number ID PQ740513 (1).

**Table 2:** Physico-chemical parameters of breeding places of malaria mosquitoes of the Crimean Peninsula and the Black Sea coast of the Caucasus.

No.	Location/Breeding Place	Date of Sampling	Density of Larvae (1–4 Instars/sq. m)	Ecological Characteristics of Habitats				
				h (m)	pH	T (°C)	ppt	O_2_ (mg/L)
	***Crimean Peninsula***							
1	Pirogovka village, Nakhimov district of Sevastopol/water storage	11.09.2016	45	63	8.05	30.0	0.34	6.0
2	Bakhchisaray town/pond	10.09.2016	28	160	8.05	18.0	0.59	7.7
3	Bakhchisaray town/dried-up creek	10.09.2016	160	160	7.70	20.7	0.70	4.5
4	Simferopol city, botanical garden/pond	10.09.2016	76	255	6.48	22.6	0.16	4.5
5	Mazanka village, Simferopol district/pond	12.07.2016	-	298	8.15	24.5	0.26	7.0
6	Konstantinovka village, Simferopol district/lake	20.08.2016	3	421	7.84	24.8	2.56	-
7	Mramornoye village, Simferopol district/lake	20.06.2016	7	493	7.62	16.5	2.14	-
8	Mezhgorye village, Belogorsky district/river	20.06.2016	9	385	7.22	21.6	1.21	-
9	Krasnosyolovka village, Belogorsky district/river spill	20.06.2016	2	401	7.62	18.3	1.94	-
10	Tylovoye village, Balaklava district of Sevastopol/pond	13.09.2016	15	295	8.15	22.1	0.22	7.8
11	Rodnikovoye village, Balaklava district of Sevastopol/puddle	20.07.2016	3	657	7.32	32.3	0.14	-
12	Rodnikovoye village, Balaklava district of Sevastopol/tree hollow	20.07.2016	7	434	7.14	24.7	0.04	-
13	Simeiz Settlement, Yalta District/mountain puddle	20.06.2016	5	149	8.32	18.0	0.17	-
14	Yalta district/forest puddle	20.07.2016	7	212	7.24	19.7	0.15	-
15	Gaspra settlement, Yalta District/water in rock cracks	20.06.2016	3	24	7.52	24.8	0.31	-
16	Gaspra settlement, Yalta district/tree hollow	20.06.2016	2	338	7.16	25.1	0.03	-
17	Voskhod settlement, Yalta district/lake	20.08.2016	8	330	7.30	21.5	0.24	-
18	Nikitsky Botanical Gardens, Yalta district/pond	13.09.2016	15	110	7.37	20.2	0.31	7.5
19	Krasnokamenka village, Yalta district/forest puddle	20.06.2016	2	770	7.23	14.9	0.11	-
20	Zaprudnoye village, Alushta district/lake	20.08.2016	14	614	7.14	17.5	0.21	-
21	Nizhnyaya Kutuzovka village, Alushta district/pond	14.09.2016	78	153	7.29	25.2	0.20	10.2
22	Alushta district/pond	14.09.2016	70	70	7.05	22.4	0.98	4.4
23	Gromovka village, Sudak district/pond	20.06.2016	3	157	7.52	16.5	2.08	-
24	Voron village, Sudak district/spring	20.08.2016	3	229	7.72	16.7	0.74	-
25	Veseloye village, Sudak district/water reserve	15.09.2016	-	131	7.28	23.2	0.81	2.3
26	Sudak district/pond	15.09.2016	-	125	8.31	22.6	0.25	-
27	Veseloye village, Sudak district/lake	20.08.2016	2	101	7.42	23.7	0.24	-
28	Dachnoye village, Sudak district/river spill	20.08.2016	-	76	7.47	22.4	3.02	-
29	Dachnoye village, Sudak district/lake	20.08.2016	4	239	7.54	23.8	0.92	-
30	Mindalnoye village, Sudak district/lake	20.07.2016	3	33	8.24	26.3	1.18	-
31	Grushevka village, Sudak district/lake	15.09.2016	29	223	6.93	22.0	0.27	8.0
32	Feodosia city/pond	16.09.2016	26	20	7.14	24.3	1.46	16.5
	***Black Sea coast of the Caucasus***							
33	Krasnogvardeyskoye village, Stavropol Krai/dried up river	12.08.2015	1	60	9.10	28.0	5.99	11.3
34	Stavropol city/pond	12.08.2015	-	484	8.85	26.5	0.20	7.9
35	Malevanyi settlement, Krasnodar Krai/river	21.08.2015	1	40	8.00	24.0	1.58	6.9
36	Razdolnaya village, Korenovsky district, Krasnodar Krai/lake	01.08.2024	10	47	8.06	25.6	1.01	10.0
37	Shengzhiy settlement, Republic of Adygeya/channel	05.08.2009	-	44	7.00	33.3	0.81	-
38	Novonikolayevskaya village, Krasnodar Krai/pond	04.08.2019	29	2	8.50	25.5	0.19	8.0
39	Tamanskoye Settlement, Temryuksky district, Krasnodar Region/lake	07.07.2016	11	155	8.00	20.5	-	8.0
40	Abinsk town, Krasnodar Krai/pond	14.08.2018	-	29	7.60	30.0	0.27	5.0
41	Gaiduk village, Novorossiysk district, Krasnodar Krai/pond	03.08.2018	16	98	7.40	26.2	0.27	4.0
42	Novorossiysk District, Krasnodar Krai/water storage	09.07.2016	14	162	8.40	21.5	-	5.0
43	Pshada village, Gelendzhik District, Krasnodar Krai/river	19.08.2015	56	27	7.30	21.8	0.48	8.1
44	Community Zarya, Tuapse district, Krasnodar Krai/car tire	31.07.2021	-	172	5.50	26.5	1.45	2.5
45	Novomikhailovsky settlement, Tuapse district, Krasnodar Krai/drainage ditch	18.08.2015	32	5	7.54	23.4	0.45	2.5
46	Agui-Shapsug village, Tuapse district, Krasnodar Krai/river	11.07.2016	67	31	7.80	22.0	-	8.0
47	Zubova Shchel village, Sochi district, Krasnodar Krai/river	13.07.2016	72	175	7.80	24.0	-	8.0
48	Sochi city, Krasnodar Krai/tree hollow	30.07.2021	-	74	6.00	24.0	2.04	3.0
49	Adler town, Krasnodar Krai/ swamp	17.07.2016	1,5	45	8.20	-	-	6
50	Verkhneveseloye village, Sochi district, Krasnodar Krai/drainage ditch	04.08.2024	3	31	9.26	27.8	0.54	7.6
51	Sochi city, Krasnodar Krai/stream	07.08.2024	-	14	7.60	-	-	-
52	Sirius settlement, Krasnodar Krai/fire pond	16.07.2016	18	15	8.40	27.0	-	4
53	Vesyoloye microdistrict, Sochi city, Krasnodar Krai/car tire	11.08.2018	-	13	7.40	27.3	0.25	-
54	Krasnaya Polyana Resort, Sochi district, Krasnodar Krai/hollow tree	25.07.2021	-	1693	5.50	22.6	2.30	3.5
55	Rosa Khutor resort, Sochi district, Krasnodar Krai/hollow tree	29.07.2021	-	1708	5.20	21.5	1.50	3.0
56	Ritsinsky National Park, Gudauta district, Abkhazia/car tire	24.07.2021	-	1041	6.00	21.3	2.78	2.5

No., location number; h (m), altitude above sea level in meters; pH, hydrogen index; T (°C), water temperature in Celsius degrees; ppt, total dissolved solids in grams per liter (parts per thousand); O_2_ (mg/L), quantity of dissolved oxygen in water.

**Table 3:** The frequencies of chromosomal variants in populations of *An. daciae* on the Crimean Peninsula and the Black Sea coast of the Caucasus.

Inversion Homo- and Heterozygotes	Frequencies of Chromosomal Variants, f ± Sf, %			
	Crimean Peninsula	Black Sea Coast of the Caucasus		
	Location 10	Location 36	Location 40	Location 42
Males, n	30	16	49	46
XL_0_	73.3 ± 8.1	56.2 ± 12.4	61.2 ± 7.0	37.0 ± 7.1
XL_1_	26.7 ± 8.1	43.8 ± 12.4	38.8 ± 7.0	63.0 ± 7.1
Females, n	36	14	59	61
XL_00_	41.7 ± 8.2	35.7 ± 12.8	32.2 ± 6.1	23.0 ± 5.4
XL_01_	50.0 ± 8.3	42.9 ± 13.2	45.8 ± 6.5	36.0 ± 6.1
XL_11_	8.3 ± 4.6	21.4 ± 11.0	22.0 ± 5.4	41.0 ± 6.3
Both sexes, n	66	30	108	107
2R_00_	100	100	100	99.1 ± 0.9
2R_05_	0	0	0	0.9 ± 0.9
2L_00_	100	100	100	100
3R_00_	100	90.0 ± 5.5	92.6 ± 2.5	91.6 ± 2.7
3R_01_	0	10.0 ± 5.5	7.4 ± 2.5	7.5 ± 2.5
3R_11_	0	0	0	0.9 ± 0.9
3L_00_	98.5 ± 1.5	83.3 ± 6.8	89.8 ± 2.9	81.3 ± 3.8
3L_01_	1.5 ± 1.5	13.3 ± 6.2	10.2 ± 2.9	15.9 ± 3.5
3L_11_	0	3.3 ± 3.3	0	2.8 ± 1.6

## Data Availability

All of the data are available in the text, figures, and tables of this article. The Sanger sequence data from *ITS2* for individual mosquitoes from Mazanka, Crimea (Table 1, location 5), are available in GeneBank [69] under accession numbers PQ510968–PQ511024; from Tylovoye, Crimea (Table 1, location 10B and 10D)—numbers PQ554994–PQ555014 and PQ550752–PQ550804; from Abinsk, Krasnodar Krai (Table 1, location 40)—numbers PQ526514–PQ526598. *COI* sequence data for individual mosquitoes from Verkhneveseloye Krasnodar Krai (Table 1, location 50) are available in GeneBank under access numbers ID PQ740514–PQ740518; from Mazanka, Crimea (Table 1, location 51)—number PQ740513.

## Abbreviations

The following abbreviations are used in this manuscript:

BRFFR	the Belarusian Republican Foundation for Basic Research
ITS2	the second internal transcribed spacer
PCR	polymerase chain reaction
RFLP	restriction fragment length polymorphism
RSF	the Russian Science Foundation
TBE	Tris–Borate–EDTA buffer
